# Pharmacological enhancement of anti-inflammatory drug efficacy through solid lipid nanoparticles in osteoarthritis and rheumatoid arthritis: a review

**DOI:** 10.1007/s10787-026-02205-6

**Published:** 2026-03-23

**Authors:** Manoj Madanahalli Ramesh, Richard Lobo, Annegowda Hardur Venkatappa

**Affiliations:** 1 Department of Pharmacognosy, Manipal College of Pharmaceutical Sciences, Manipal Academy of Higher Education, Manipal, India; 2 Department of Pharmacognosy, Sri Adichunchanagiri College of Pharmacy, Adichunchanagiri University, B. G Nagara, Mandya, Karnataka 571448 India

**Keywords:** Nanomedicine, Drug delivery, Pharmacokinetics, Controlled release, Regulatory considerations, Patent landscape

## Abstract

Chronic inflammatory diseases, such as rheumatoid arthritis and osteoarthritis, are sustained by dysregulated cytokine signaling, eicosanoid production, and the continuous activation of intracellular inflammatory pathways. Although nonsteroidal anti-inflammatory drugs, corticosteroids, and disease-modifying drugs are foundational treatments, their long-term use is limited due to unfavorable pharmacokinetics, lack of tissue selectivity, and systemic side effects. Solid lipid nanoparticles (SLNs) have emerged as a promising delivery system. They serve as lipid carriers, enabling better control of drug distribution while preserving pharmacodynamic effects. Preclinical studies indicate that SLN formulations can enhance the pharmacokinetics of anti-inflammatory drugs by promoting local retention, providing extended release, and increasing local drug exposure at sites of inflammation following topical, intra-articular, or systemic administration. At the molecular level, drugs delivered using SLNs consistently inhibit key inflammatory mediators, including TNF-α, IL-1β, IL-6, and PGE₂. These actions are associated with reduced activation of nuclear factor-κB and Janus kinase/signal transducer and activator of transcription pathways, as well as decreased oxidative stress and leukocyte accumulation in inflamed joints and surrounding tissues. Importantly, SLNs do not change the inherent action of the drugs they carry; instead, they enhance their pharmacological effectiveness by prolonging drug concentration at the target site and reducing exposure to non-target tissues. This fine-tuning of pharmacokinetics can lead to dose-saving strategies, which are particularly vital in the long-term management of arthritis. SLNs are an active delivery system that improves the performance of anti-inflammatory drugs by modulating controlled exposure and altering molecular pathways. However, further prolonged dosing studies and clinical trials are necessary to fully establish their clinical utility.

**Graphical abstract:**

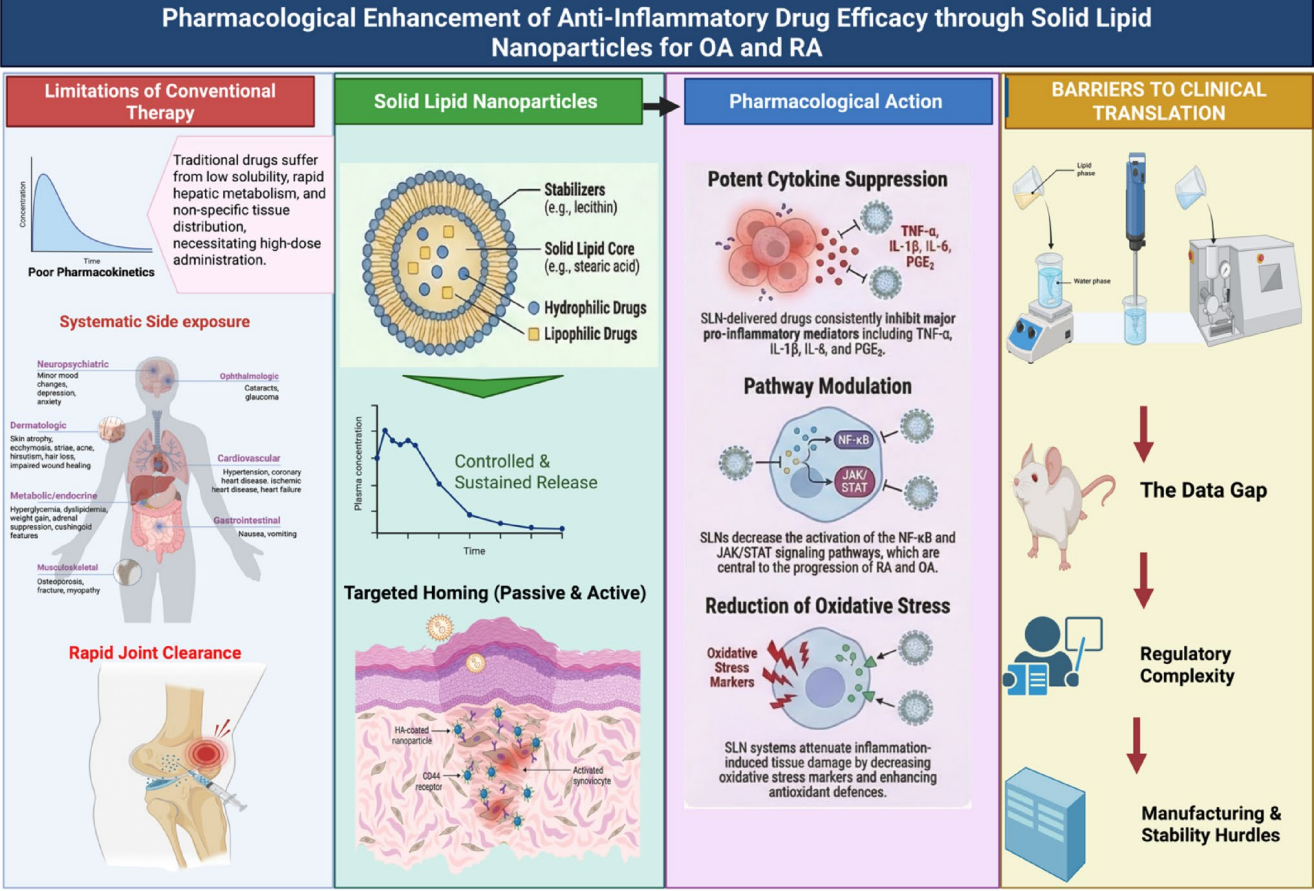

## Introduction

Inflammation is a fundamental physiological process that responds to injury or infection through a multi-cellular, molecularly complex series of pathways that restore tissue homeostasis. Acute inflammation is an essential protective response, but its lack of control or excessive persistance can lead to a myriad of disabling conditions that have an enormous impact on public health and quality of life worldwide. Inflammatory diseases such as rheumatoid arthritis (RA) and osteoarthritis (OA) are chronic disorders that exhibit prolonged immune response, tissue destruction, and functional limitation. RA is a systemic autoimmune disease characterized by chronic inflammation of synovial joints, leading to progressive cartilage and bone destruction and causing pain and disability (McInnes and Schett 2011; Chuang et al. 2018). OA is the most common form of arthritis and is no longer regarded simply as a ‘wear and tear’ disease, but is increasingly understood as a complex disorder with an important inflammatory component, characterised by degradation of articular cartilage and subchondral bone remodeling, often worsened by low-grade systemic inflammation (Pap and Korb-Pap 2015).

The current therapeutic arsenal for the treatment of chronic inflammatory diseases consists mainly of non-steroidal anti-inflammatory drugs (NSAIDs), corticosteroids, and disease-modifying anti-rheumatic drugs (DMARDs) such as biological agents. Although these traditional approaches may provide symptomatic benefit and sometimes delay the progression of disease, they are often limited in terms of efficacy and safety profile. As an example, NSAIDs are commonly used for their analgesic and anti-inflammatory effects, but are notorious for causing gastrointestinal toxicities, renal dysfunction, and cardiovascular events, especially when they are taken chronically. Corticosteroids, being powerful anti-inflammatory drugs, are successful in acute attacks; however, long-term systemic use leads to a range of side effects, including metabolic aberrations, immunosuppression, and osteoporosis (Rhen and Cidlowski 2005; Singh and Choudhary 2025). Although conventional DMARDs can be efficacious at slowing disease progression, they generally have a slow onset of action and may cause systemic side effects that are potentially severe, with a need for monitoring patients closely. Moreover, most anti-inflammatory drugs have poor pharmacokinetic and biodistribution properties, such as low solubility, rapid metabolism, and non-specific tissue distribution, which reduce their therapeutic index and often necessitate high-dose administration to achieve adequate concentrations at the inflamed site, with an increased risk of off-target effects (Singh and Choudhary 2025).

The disadvantages of traditional anti-inflammatory drugs have driven intense research into new drug delivery systems to improve their therapeutic efficacy and reduce toxic side effects. Of these, lipid-based nanoparticles have proven particularly effective for the better delivery of anti-inflammatory drugs. Among those, SLNs are regarded as a second-generation colloidal carrier system composed of solid lipids at body temperature and offer several advantages over conventional formulations. SLNs can entrap hydrophilic and lipophilic drugs within the particles, preventing biodegradation and providing sustained release (Chuang et al. 2018; Duong et al. 2020; Singh and Choudhary 2025). Their small size is favorable for the enhanced permeation and retention effect at the site of inflammation, which permits passive targeting. Furthermore, through surface modification, nanoparticles can be actively targeted to specific cells or receptors involved in the inflammation cascade. This targeted therapy would result in a substantial enhancement of the selectivity of anti-inflammatory drugs for inflamed cells and tissues over noninflamed tissues, with less systemic exposure, thereby improving the treatment ratio substantially. Studies have also shown that lipid-based nanoparticles could enhance the therapeutic benefits and diminish the side effects of anti-arthritic compounds for patients. For instance, SLNs have been examined for long-term drug delivery via transdermal application of anti-inflammatory drugs such as piroxicam (Chuang et al. 2018; Borges et al. 2020; Lakshmi and Ashok Kumar 2024; Singh and Choudhary 2025). Additionally, SLNs have the potential to deliver complex anti-inflammatory agents, such as small interfering RNA (siRNA), for the treatment of chronic inflammatory diseases like RA and OA by suppressing pro-inflammatory cytokines, such as tumor necrosis factor-α (TNF-α). The co-encapsulation of glucocorticoids, such as betamethasone acetate, with siRNA within the SLNs also helps decrease the pro-inflammatory activity of the nanoparticles, thereby increasing their therapeutic efficacy (Albuquerque et al. 2015).

This review explores how SLNs enhance the effectiveness of anti-inflammatory drugs. The pharmacological properties of SLNs that improve drug delivery, including increased bioavailability, prolonged drug release, and targeted deposition at sites of inflammation, are discussed. The therapeutic potential of SLN formulations for various chronic inflammatory diseases is examined, with particular emphasis on their application in the treatment of arthritis. This review aims to highlight the promise of SLNs in advancing traditional anti-inflammatory therapies and to provide insights into future directions that may lead to more efficient and safer treatment strategies for patients with chronic inflammation. In addition, recent advances in this field are compiled to establish a foundation for future research and clinical translation.

## Pharmacological rationale for solid lipid nanoparticles in inflammation

**Fig. 1:**
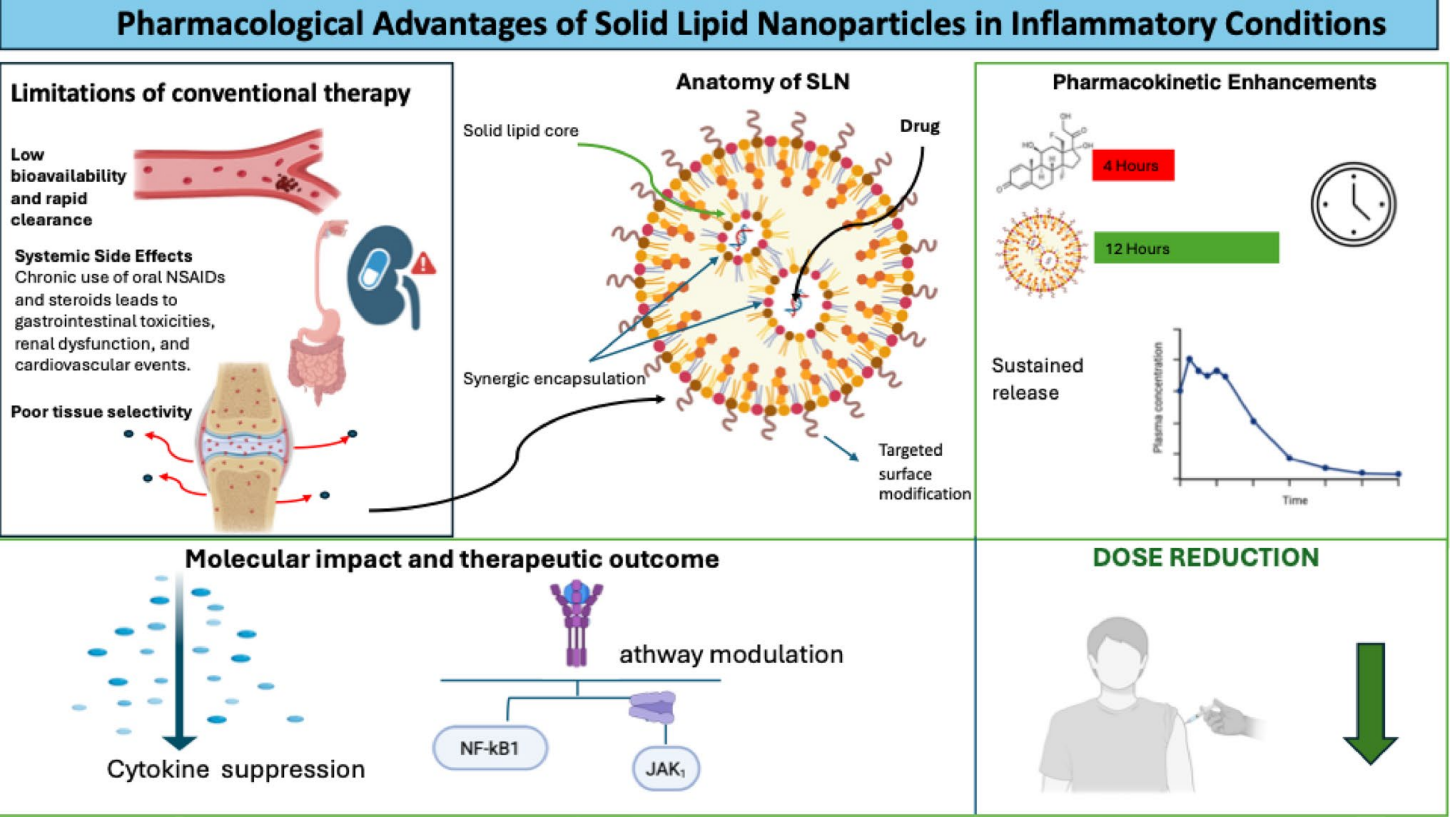
Pharmacological advantages of solid lipid nanoparticles in inflammatory conditions. SLNs enhance the therapeutic performance of anti-inflammatory agents by improving pharmacokinetic and pharmacodynamic properties, including increased bioavailability and sustained drug release. The solid lipid core enables efficient encapsulation and protection of anti-inflammatory drugs, while facilitating targeted accumulation in inflamed tissues. SLNs also support co-delivery of multiple therapeutic agents, such as anti-inflammatory drugs and nucleic acid–based modulators, resulting in synergistic suppression of inflammatory mediators including TNF-α and IL-1β. By reducing systemic exposure and minimizing off-target effects, SLN-based delivery improves the therapeutic index and safety profile of anti-inflammatory pharmacotherapy, particularly in chronic inflammatory diseases such as osteoarthritis and rheumatoid arthritis (Dianzani et al. 2017; Peng et al. 2017; Chuang et al. 2018; Krishnatreyya et al. 2019).

Long-term pharmacological treatment is usually required for chronic inflammatory diseases, such as OA and RA. Nevertheless, traditional anti-inflammatory drugs often have limitations in efficacy and safety. SLNs are emerging drug delivery system to address these issues, as they have the potential by modulating absorption, distribution, and tissue retention. Traditional anti-inflammatory drugs might be faced with some pharmacological limitations such as low solubility, short half-life, and systemic side effects. The above problems usually result in suboptimal drug exposure at the target site, requiring a higher dose that increases the risk of side effects and narrows the therapeutic index (Dianzani et al. 2017; Krishnatreyya et al. 2019). SLN are designed to enhance the delivery of drugs, especially in inflammatory states. Their specific chemical composition, consisting mainly of a solid lipid form and lecithin, offers several advantages. Figure 1 illustrates the advantages of solid lipid nanoparticles in inflammatory diseases.

## Pharmacokinetic modulation of anti-inflammatory drugs by SLNs

SLNs consistently reshape the pharmacokinetic (PK) behavior of anti-inflammatory drugs used in the long-term management of OA and RA. Across topical and systemic routes, SLNs enhance drug exposure by improving absorption and reducing pre-systemic loss, leading to higher and more durable tissue concentrations. For instance, Bhaskar et al. (2009) reported that lipid-based flurbiprofen gels achieved greater exposure than oral free drug, while Liu et al. (2010) demonstrated enhanced dermal localization of diclofenac delivered via SLNs. These pharmacokinetic gains translate into sustained therapeutic coverage with reduced dosing frequency.

**Fig. 2:**
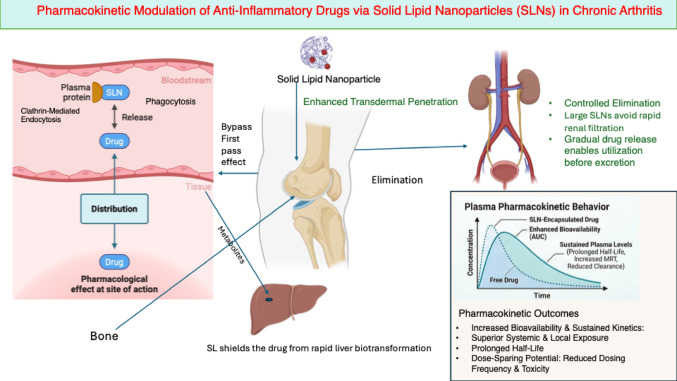
Schematic representation of pharmacokinetic modulation of anti-inflammatory drugs by SLNs in chronic arthritis. SLNs enhance absorption, promote inflammation-biased tissue distribution, prolong systemic and local residence time, protect drugs from rapid hepatic metabolism, and enable controlled elimination. These effects collectively increase bioavailability, extend half-life, improve mean residence time, and support dose-sparing strategies for long-term management of osteoarthritis and rheumatoid arthritis

A defining advantage of SLN formulations is their ability to provide sustained and controlled drug release. Bhatia and Gupta (2016) showed that ketorolac-loaded SLN gels released substantially less drug over 24 h compared with conventional gels, whereas Shinde et al. (2016) reported that intra-articular lipid carriers of methotrexate maintained joint-site drug release for approximately 108 h. Such smoothing of release kinetics minimizes peak–trough fluctuations and supports extended dosing intervals, which is particularly advantageous for chronic inflammatory diseases. As illustrated in Fig. 2, solid lipid nanoparticles modulate key pharmacokinetic processes, including absorption, tissue distribution, metabolic protection, and elimination, thereby enhancing bioavailability and prolonging drug residence in inflamed joints.

SLNs further optimize tissue distribution and drug residence. Zhou et al. (2018) demonstrated that hyaluronic acid–coated prednisolone SLNs preferentially accumulated in inflamed joints and persisted longer in systemic circulation. Similarly, Krishnatreyya et al. (2019) showed that topical piroxicam SLNs enhanced dermal penetration and retention, creating peri-articular drug depots compatible with reduced application frequency. At the systemic level, Zheng et al. (2024) reported that albumin–lipid dexamethasone palmitate nanoparticles prolonged plasma detectability to approximately 12 h, compared with about 4 h for the free drug. Collectively, the mechanistic evidence summarized in Table 1 demonstrates that SLNs function as active pharmacological enhancers.

**Table 1:** Pharmacokinetic Effects of Solid Lipid Nanoparticles in Arthritis-Related Studies (PK-only)

First author (Year)	Drug / bioactive	Route of administration	Pharmacokinetic parameter influenced	Observed PK change with SLN	PK relevance to OA / RA therapy
Bhaskar et al. (2009)	Flurbiprofen	Transdermal (lipid gel)	Bioavailability (area under the plasma concentration–time curve [AUC] and maximum plasma concentration [Cmax]); exposure duration	↑C_max/AUC vs. oral free drug; sustained exposure over 24 h	Higher exposure with flatter profile → reduced dosing frequency
Liu et al. (2010)	Diclofenac sodium	Topical (SLN)	Absorption; skin retention; release kinetics	Biphasic → sustained release; increased dermal localization vs. solution	Prolonged local availability with fewer applications
Krishnatreyya et al. (2019)	Piroxicam	Topical (SLN gel)	Dermal penetration; local retention	Enhanced skin permeation and sustained permeation vs. free-drug gel	Extended peri-articular reservoir→ longer application intervals
Bhatia and Gupta (2016)	Ketorolac	Transdermal (SLN gel)	Release duration	~ 55% released at 24 h (SLN) vs. ~86% (conventional gel)	Smoother exposure enables extended dosing intervals
Shinde et al. (2016)	Methotrexate (lipid carrier)	Intra-articular (gel)	Release duration; joint residence	Prolonged release ~ 108 h free drug	Improved joint exposure; decreased injection frequency
Zhou et al. (2018)	Prednisolone (HA-SLN)	Intravenous	Circulation time; joint retention	Joint-selective accumulation; longer persistence vs. free or non-targeted SLNs	Better joint PK with minimized systemic exposure
Zheng et al. (2024)	Dexamethasone palmitate (albumin–lipid NP)	Intravenous	Half-life; systemic exposure	Detectable ≤ 12 h vs. ≤4 h for free drug	Less frequent dosing during chronic management

## Pharmacological mechanisms modulated by SLNs

SLNs can enhance the efficacy of anti-inflammatory drugs through multiple mechanisms, including modulation of inflammatory signaling pathways, improved tissue-targeted drug accumulation, and sustained drug activity. The summary in Table 2 indicates that SLN formulations have consistently inhibited the expression of major pro-inflammatory mediators after drug dosing across different classes, including corticosteroids, NSAIDs, kinase inhibitors (e.g., Lapatinib), and natural bioactives.

A leading paradigm is the suppression of central cytokines, specifically Tumor Necrosis Factor alpha (TNF-α), interleukin-1β (IL-1β) and interleukin-6 (IL-6) that are responsible for chronic inflammatory diseases such as rheumatoid arthritis and osteoarthritis. Hyaluronic acid–coated prednisolone SLNs outperformed free prednisolone and effectively suppressed cytokines in the joint, highlighting the significance of tissue-targeted nanoparticles. Co-delivery of dexamethasone–cholesteryl butyrate and betamethasone–TNF-α siRNA via SLNs induced additive or synergistic suppression of cytokine signal transduction, respectively. The anti-edematous activity of NSAID-loaded SLNs is mainly due to modulation of the cyclooxygenase-2 (COX-2)/prostaglandin E₂ (PGE₂) axis, resulting in a greater and longer-lasting effect than that observed with traditional preparations. Piroxicam, ibuprofen, and ketorolac SLNs exhibited sustained local effects and enhanced penetration into the skin, which favours their potential use for chronic musculoskeletal pain control. In addition, upstream inflammatory signaling pathways are well-controlled. NF-κB activation was repressed by curcumin, cannabidiol, and fluticasone SLNs; tofacitinib SLNs potently potentiated the inhibition of Janus kinase/signal transducer and activator of transcription (JAK/STAT)-dependent responses. Concurrently, some SLN systems decreased levels of oxidative stress markers and enhanced antioxidant defense mechanisms, thereby attenuating inflammation-induced tissue damage.

**Table 2:** Pharmacological mechanisms modulated by SLN

First author (Year)	Drug / Bioactive encapsulated	SLN composition (lipid matrix & surfactant)	Targeted inflammatory pathway(s)	Key inflammatory biomarkers measured	Experimental model (cell line / animal / disease model)	Mechanistic outcome of SLN delivery	Pharmacological advantage vs. free drug	Relevance to chronic inflammation / osteoarthritis
Zhou et al. (2018)	Prednisolone (glucocorticoid)	Lipid matrix: Glyceryl monostearate (GMS), soy phosphatidylcholine (SPC), and cholesterol with didodecyl dimethyl ammonium bromide (DDAB); surface functionalization: hyaluronic acid (HA).	Cytokine axis (TNF-α, IL-1β, IL-6); joint-homing via HA–CD44	Serum TNF-α, IL-1β, IL-6	In vivo: Collagen-induced arthritis (CIA) in mice; RAW264.7 in vitro	HA-SLNs/PD accumulated in inflamed joints, reduced systemic cytokines, preserved bone/cartilage vs. controls	Greater joint accumulation, prolonged circulation; superior efficacyto free PD and non-HA SLNs	High—direct RA model; joint targeting relevant to chronic arthritis and OA pathology
Dong et al. (2024)	Dexamethasone + cholesteryl butyrate (DxCb-SLN)	Lipid matrix: cholesteryl butyrate (lecithin/phospholipid surfactants; 2-phenylethanol co-solvent)	NF-κB modulation; cytokines (TNF-α, IL-1β)	Plasma TNF-α, IL-1β; PBMC cytokine secretion	In vivo: DSS-induced colitis (mice); in vitro PBMCs	SLNs lowered TNF-α > IL-1β, inhibited inflammatory cell adhesion; additive steroid–butyrate effect	Higher potencyat lower doses vs. free drugs; time-protected release	Chronic intestinal inflammation (IBD) model; mechanistic NF-κB control relevant to chronic inflammatory states
De Melo et al. (2016)	15-deoxy-Δ12,14-prostaglandin J2 (15d-PGJ2; PPAR-γ agonist)	Matrix: tripalmitin; Surfactant: Poly Vinyl Alcohol	Cytokines (↑ IL-10, ↓ IL-1β, IL-17); neutrophil trafficking	IL-1β, IL-17, IL-10(peritoneal fluid)	In vivo: carrageenan, LPS, mBSA murine models; in vitro 3T3 cells	SLNs reduced neutrophil migration; re-balanced cytokines (↑IL-10/↓IL-1β, IL-17)	~ 100-fold lower dose needed vs. free 15d-PGJ2; controlled release, high EE (~ 96%)	Broad anti-inflammatory immunomodulation applicable to chronic arthritis; dose-sparing benefits for long-term use
Peng et al. (2017)	Piroxicam (NSAID)	Matrix: glycerol monostearate + lecithin; Surfactant: Tween-80 (0.75% w/w)	COX-2/PGE₂ axis; neutrophil-driven edema	PGE₂ (paw tissue ELISA); paw volume	In vivo: Carrageenan-induced rat paw edema; ex vivo skin permeation	SLNs decreased PGE₂ and edema vs. comparators; enhanced transdermal flux	Sustained release + deeper skin penetration; mitigates GI toxicity risks of oral piroxicam	Relevant to chronic musculoskeletal pain (OA/RA flares) via sustained local COX-2 control
Krishnatreyya et al. (2019)	Piroxicam (NSAID)	Matrix: stearic acid; Surfactants: Pluronic F68 + phospholipon 80	Prostaglandin-mediated edema (COX pathway, inferred by model)	Edema inhibition; permeation metrics	In vivo: Carrageenan rat paw edema; in vitro/ex vivo release & permeation	Sustained dermal permeation and prolonged anti-edema effect vs. gel	Longer residence time at inflamed site; safe topical alternative	Chronic joint pain control through depot-like dermal delivery in OA/RA
Bhatia and Gupta (2016)	Ketorolac (NSAID)	Matrix: stearic acid; Surfactant: soya lecithin	COX-mediated edema (model-based)	Paw edema inhibition time-course	In vivo: Carrageenan rat paw model	SLN gel produced higher and longer-lasting inhibition (to 24 h) than conventional gel	Prolonged effect (sustainrelease) and improved skin retention	Applicable to chronic pain management where frequent dosing is undesirable (e.g., OA)
Bagde et al. (2019)	Ibuprofen (NSAID)	Matrix: Compritol ATO 888 / Precirol ATO 5 / GMS / stearic acid (screened); Surfactant: Kolliphor RH40	COX-mediated edema	% edema inhibition; skin deposition	In vivo: Carrageenan rat paw; topical gel	SLN gel doubled anti-edema effect vs. control gel; ~3× skin deposition	Higher release + deposition (84% release; ↑local exposure) vs. free gel	Supports chronic local therapy with reduced systemic exposure
Desoqi et al. (2021)	Fluticasone propionate (corticosteroid)	Matrix: stearic acid; Surfactant: Tween-80	Cytokines (TNF-α), PGE₂	TNF-α, PGE₂	In vivo: Carrageenan-induced inflammation; topical gel	SLNs reduced TNF-α (− 61%) & PGE₂ (− 51.5%), outperforming marketed cream	Improved topical PD without higher systemic load; stability & skin retention ↑	Chronic dermatoses; corticosteroid-sparing topical strategy for persistent inflammation
Gaur et al. (2016)	Curcumin (natural anti-inflammatory)	Matrix: GMS + stearic acid + ceramide-2:palmitic acid; Surfactant: Tween-80	Oxidative stress & cytokines (model-based); transdermal targeting	Edema inhibition; PK (bioavailability ↑ to ~ 68%)	In vivo: Carrageenan edema (rats); ex vivohuman skin permeation	SLNs achieved 90.8% edema inhibition (6 h); markedly ↑ skin permeation and systemic exposure	Major PK gain(bioavailability ↑) and fast dermal uptake	Relevant to chronic OA pain (topical curcumin with meaningful tissue exposure)
Sharma et al. (2019)	Curcumin (natural)	Matrix: binary solid lipids—stearic acid + tristearin; Surfactants: Pluronic F68 (+ PVA)	NF-κB inactivation; cytokines & oxidative stress	TNF-α, MPO; lipid peroxidation, protein carbonyls	In vivo: DSS colitis (oral C-SBLNs)	NF-κB pathway dampened; ↓ TNF-α, MPO, oxidative injury; preserved colonic structure	Sustained release (24 h), enhanced uptake at inflamed tissue vs. free curcumin	Models chronic gut inflammation; NF-κB control is central in chronic inflammatory disorders
Alcantara et al. (2024)	Cannabidiol (CBD; natural)	Matrix: GMS; Surfactants: poloxamers (stabilization)	NF-κB–driven cytokines; oxidative stress/Reactive oxygen species (ROS)	TNF-α, IL-6, IL-1β; ROS/RNS (cell models)	In vitro: RAW 264.7 macrophages; SW1353 chondrocytes (OA-relevant)	CBD-SLNs reduced cytokines and ROS > free CBD; sustained release	Improved bioavailability & stability with controlled release	Direct OA relevance (chondrocyte model); fits chronic joint inflammation paradigm
O’Mary et al. (2019)	Betamethasone acetate + TNF-α siRNA(co-encapsulated)	Matrix: DOTAP + lecithin + cholesterol; PEG-hydrazone-stearate(PHC)	TNF-α suppression; NF-κB (GC–GR interaction)	TNF-α, IL-1β, IL-6, MCP-1	In vitro: J774A.1 macrophages; in vivo: BALB/c mice (i.v.)	Optimal BA: siRNA ratio minimized cytokine surge; glucocorticoid co-delivery blunted NP-induced inflammation	Lower inflammatory toxicity vs. free components; single-system dosing	Platform relevance for chronic inflammatory states where TNF-α is central (e.g., RA)
Roy et al. (2023)	Tofacitinib citrate (JAK inhibitor)	Matrix: tripalmitin-based SLNs; Surfactant: Brij-35; (NTC as release retardant)	JAK/STAT down-stream cytokines (functional readout); edema	Paw diameter (CFA model)	In vivo: CFA-induced inflammation (rats), topical gel	SLN-TFC gel normalized paw swelling over 21 days > marketed gel	Prolonged action & permeability enhancement vs. comparator	JAK inhibition in chronic arthritis; sustained topical control aligns with OA/RA needs

GMS (glyceryl monostearate); SPC (soy phosphatidylcholine); DDAB (didodecyldimethylammonium bromide); CD44 (cluster of differentiation 44); PBMCs (peripheral blood mononuclear cells); CIA (collagen-induced arthritis); DSS (dextran sulfate sodium); IBD (inflammatory bowel disease); PPAR-γ (peroxisome proliferator-activated receptor gamma); LPS (lipopolysaccharide); mBSA (methylated bovine serum albumin); ELISA (enzyme-linked immunosorbent assay); MPO (myeloperoxidase); ROS (reactive oxygen species); RNS (reactive nitrogen species); DOTAP (1,2-dioleoyl-3-trimethylammonium-propane); PEG (polyethylene glycol); GC (glucocorticoid); GR (glucocorticoid receptor); CFA (complete Freund’s adjuvant)

## Safety, toxicity, and translational considerations of SLNs

The preclinical development of SLNs for anti-inflammatory therapy illustrates a promising safety record based on the inherent biocompatibility and biodegradability of their lipid matrices. Similar to the physiological lipids used, glyceryl monostearate, stearic acid, triglycerides, and phospholipid stabilizers are used in multiple SLN formulations and exhibit low acute in vitro and in vivo toxicity (Bhatia and Gupta 2016; Peng et al. 2017; Bagde et al. 2019). Other surfactants, such as Tween-80, Poloxamers, Lecithins, and Phosphatidylcholines, are also extensively used and are known to be safe. These materials then tend to be good with regard to hemocompatibility, and low cytotoxicity at formulation-relevant concentrations, as is the case with these curcumin SLNs (Gaur et al. 2016), piroxicam SLNs (Krishnatreyya et al. 2019), and ibuprofen SLNs (Bagde et al. 2019). At best, undesirable toxicity does not appear unless at supra-therapeutic exposures or long-term in vitro incubation (that have less physiologic excipients as in cationic lipids e.g., DOTAP, or high-PVA content), which emphasizes formulation optimization in safety (De Melo et al. 2016; O’Mary et al. 2019).

One aspect that recurs throughout preclinical work is the diminishing of systemic toxicity compared to standard systemic delivery of NSAIDs or corticosteroids. The vast majority of SLN formulations are dose-sparing due to effective delivery at the site of action, enabling efficacy at lower systemic exposure. For example, 15-deoxy-Δ12,14-prostaglandin J₂. SLNs exhibited very strong anti-inflammatory properties at doses that were nearly 100-fold lower than the free drug, highlighting the advantage of pharmacodynamic efficiency obtained with encapsulation in nanoparticles (De Melo et al. 2016). Piroxicam, ketorolac, and ibuprofen topical NSAID SLN gels demonstrate increased permeability through the skin, decreased systemic absorption, and prolonged local effects: factors that reduce gastrointestinal and renal toxicities associated with chronic NSAID therapy (Bhatia and Gupta 2016; Peng et al. 2017; Bagde et al. 2019). Joint-targeted SLNs for corticosteroids such as prednisolone nanoparticles coated with HA preferentially accumulate in inflamed synovium and decrease local cytokines more effectively than free steroid, suggesting the potential of steroid-sparing therapeutic strategies in chronic diseases such as rheumatoid arthritis and osteoarthritis (Zhou et al. 2018).

However, despite these optimistic tendencies, SLNs are susceptible to the well-known nanoparticle-associated risks common with all colloidal delivery systems. The liver and spleen are the commonly identified organs in the reticuloendothelial system (RES) for particle uptake, which can cause slow accumulation of nanoparticles in the target tissues and lead to a systemic deviation of particles away from the target tissue. To some degree, this drawback can be alleviated through PEGylation and surface modifications (e.g., HA coating) (Zhou et al. 2018; Dong et al. 2024), although such approaches may raise concerns about immunogenicity or anti-PEG antibody production over the long term. Even where dose-dependent increases in hemolysis or cell stress have been shown to be formulation-dependent, some cationic lipid or high surfactant load formulations may pose a potential barrier to development and should be carefully evaluated. In addition, chronic inflammatory conditions, like osteoarthritis or inflammatory bowel disease, will necessitate longer courses of treatment, but long-term exposure information of these nanoparticles is quite scarce. Available studies are mainly limited to short (1–4 week) dosing durations and fail to thoroughly assess chronic biodistribution, nanoparticle retention, low-grade immune activation, or cumulative tissue remodelling effects (O’Mary et al. 2019, p. 1515; Sharma et al. 2019).

Additional challenges stem from the translation of SLN platforms from preclinical systems to clinical translation. SLN characteristics (particle size, polydispersity, drug loading, crystallinity) are extremely sensitive to the processing parameters, and thus, scalability and reproducibility are still a major challenge. The production of these systems can be batch-to-batch variable using manufacturing methods (e.g., hot-melt emulsification or solvent evaporation), further complicating regulatory approval. Drug expulsion from stored formulations, high water content in dispersions, and limited loading of some actives are repeatedly reported as hurdles in drug delivery or as a need for further formulation engineering. Nanoparticle-specific critical quality attributes (CQAs), such as surface chemistry, long-term stability, and nanoparticle–protein interactions, require a much more rigorous characterization than that of small-molecule drugs, which is now being demanded by regulatory agencies. Toxicology expectations also go beyond the classic biosafety profiles, necessitating nanoparticle-specific safety studies that address biodistribution, immunogenicity, RES saturation, and chronic effects. Perhaps most concerning of all, clinical evidence is lacking. The preclinical studies reviewed here all show greater efficacy and favorable safety signals compared to the free drugs; however, these models do not replicate the complexity of human chronic inflammatory disease, especially in osteoarthritis, in which joint vascularity, synovial permeability, and cartilage barrier are markedly different from rodent systems. Claims of greater safety and therapeutic index must be considered preliminary without data from trials in humans. The conclusions regarding long-term tolerability, potential for disease modification, and risk–benefit balance in human patients are limited by over-reliance on in vitro macrophage or chondrocyte assays, short-term rodent inflammation models, and acute cytokine readouts (Zhou et al. 2018; Dong et al. 2024; Alcantara et al. 2024). Key safety findings and translational limitations of SLN formulations are summarized in Table 3, emphasizing favorable short-term tolerability alongside gaps in long-term exposure data.

**Table 3:** Safety, toxicity, and translational considerations of SLN-based anti-inflammatory formulations

First author (Year)	Drug / SLN system	Route of administration	Key safety or toxicity observations	Translational relevance / limitations
Peng et al. (2017)	Piroxicam SLNs	Topical / transdermal	Low irritation, reduced systemic exposure vs. oral NSAIDs	Good candidate for chronic localized pain; long-term safety unknown
Bhatia and Gupta (2016)	Ketorolac SLNs	Topical	Sustained effect with minimal local toxicity	Reproducibility and scale-up challenges limit translation
Bagde et al. (2019)	Ibuprofen SLNs	Topical	Enhanced skin deposition, no major dermal toxicity	Potential for OA flares; requires clinical dermal safety data
Zhou et al. (2018)	HA-coated prednisolone SLNs	Intravenous	Reduced systemic steroid burden; no major organ toxicity reported	Targeted delivery promising, but long-term steroid nanoparticle exposure untested
Dong et al. (2024)	Dexamethasone–butyrate SLNs	Oral	Lower cytokine release; low acute toxicity	Colitis models encouraging; human mucosal biodistribution uncertain
De Melo et al. (2016)	15d-PGJ₂ SLNs	Subcutaneous / intraperitoneal	Effective at very low doses; mild hemolysis at high concentrations	Dose-sparing attractive; long-term immune effects unknown
O’Mary et al. (2019)	Betamethasone + TNF-α siRNA SLNs	Intravenous	Reduced nanoparticle-induced cytokine surges; steroid-related risks remain	Relevant for severe inflammation; regulatory hurdles for siRNA–steroid systems
Gaur et al. (2016)	Curcumin SLNs	Transdermal	Minimal skin toxicity; enhanced bioavailability	Natural compound appeal; human dosing and chronic tolerance unclear
Sharma et al. (2019)	Curcumin SBLNs	Oral	No mortality; reduced oxidative damage	IBD relevance strong; translation limited by GI variability
Alcantara et al. (2024)	CBD-SLNs	Oral	Reduced ROS and cytokines; no major cytotoxicity reported	OA-relevant in vitro data; lacks in vivo chronic safety studies

## Comparison between conventional formulations and solid lipid nanoparticle-based drug delivery systems

Conventional oral and parenteral formulations of NSAIDs, corticosteroids and methotrexate used in osteoarthritis and rheumatoid arthritis are constrained by low and variable bioavailability, extensive first-pass or systemic metabolism, rapid clearance from plasma and joint spaces, and broad tissue distribution that underlies gastrointestinal, renal, cardiovascular and systemic immunosuppressive toxicities. These pharmacokinetic drawbacks translate into pharmacodynamic limitations, including short duration of analgesic and anti-inflammatory action, incomplete suppression of cytokine-driven synovitis, and the need for frequent dosing or high doses that narrow the therapeutic window (Pham et al. 2020; Ling et al. 2020).

SLNs encapsulate anti-inflammatory drugs within a solid lipid core, altering absorption, distribution, metabolism and elimination by improving solubility, shielding drugs from premature degradation, and providing controlled release from sub 200 nm carriers that can accumulate in inflamed skin and synovium. For topical NSAIDs such as diclofenac, ibuprofen, piroxicam and ketorolac, SLN gels increase dermal penetration and local drug retention while limiting systemic uptake, thereby sustaining local drug concentrations over painful joints and reducing gastrointestinal exposure. For intra-articular or systemic delivery of methotrexate and prednisolone, lipid-based nanocarriers prolong joint residence and circulation time, enable passive and ligand-mediated (e.g., hyaluronic acid–CD44) targeting to inflamed synovium, and enhance suppression of cytokines and downstream signaling with lower total doses. Collectively, SLN-based systems improve pharmacokinetics and pharmacodynamics by bypassing or reducing first-pass metabolism, slowing clearance, restricting distribution to non-inflamed tissues, and mitigating systemic toxicity while providing prolonged, site-directed anti-inflammatory action and higher effective bioavailability (Bhatia and Gupta 2016; Shinde et al. 2016; Zhou et al. 2018; Pham et al. 2020; Dhule and Nandgude 2023). A comparative summary of pharmacokinetic, pharmacodynamic, and mechanistic advantages of solid lipid nanoparticles over conventional formulations in arthritis therapy is presented in Table 4.

**Table 4:** Representative comparison of conventional drug delivery systems and solid lipid nanoparticle formulations for anti-inflammatory and anti-arthritic drugs with pharmacokinetic, pharmacodynamic, and mechanistic outcome

Drug	Disease	Conventional formulation	SLN formulation	Pharmacokinetic improvement	Pharmacodynamic improvement	Mechanistic advantage	Outcome	References
Diclofenac	Osteoarthritis and musculoskeletal pain	Standard 1–3% topical gels with short half-life, high first-pass metabolism and substantial GI risk.	Diclofenac sodium-loaded SLNs in transdermal or gel systems using glyceryl monostearate or guggul lipid as matrix.	Two-step release (initial burst then slow phase) with enhanced dermal localization and lower transdermal flux than solution, indicating higher skin retention and reduced systemic	Comparable or superior pain relief to conventional diclofenac preparations with fewer systemic adverse effects due to predominantly local delivery.	Lipid matrix forms a reservoir within stratum corneum and viable epidermis, sustaining diclofenac levels over affected joints while bypassing hepatic first-pass and limiting distribution to non-inflamed organs.	Improved symptomatic control of OA pain with potential reduction in oral dose and GI toxicity when SLN-based topical therapy replaces or supplements systemic diclofenac.	Liu et al. (2010); Ling et al. (2020)
Ibuprofen	Osteoarthritis and localized joint pain	Conventional 5% ibuprofen gels show limited skin permeation and modest (~ 20%) edema inhibition in rat paw models, requiring repeated application.	Ibuprofen-loaded SLN gels (≈ 0.5%) produced by hot-melt extrusion or related methods; particles ~ 60–100 nm with high (~ 90%) entrapment.	Higher cumulative release and ~ 3-fold greater skin deposition than control gel, with more drug retained in skin and less in receptor phase, indicating stronger local exposure and reduced systemic	Approximately two-fold greater and longer-lasting inhibition of carrageenan-induced paw edema versus conventional ibuprofen gel at similar dose.	Nanoparticles enhance partitioning of ibuprofen into skin lipids and create a local depot adjacent to inflamed periarticular tissues, flattening concentration–time profiles and allowing lower applied dose for equivalent effect.	Stronger and more sustained local analgesic and anti-inflammatory action with potential for decreased dosing frequency and reduced risk of systemic NSAID adverse events.	Bagde et al. (2019); Pham et al. (2020)
Piroxicam	Chronic osteoarthritic and musculoskeletal pain	Oral piroxicam capsules and conventional gels provide effective analgesia but are linked to dose-dependent GI and renal toxicity; standard gels show relatively rapid release and limited retention.	Topical piroxicam SLN gels based on stearic acid or glycerol monostearate matrices stabilized with phospholipids and surfactants.	Enhanced dermal permeation with greater drug accumulation within skin layers and slower decline in permeation rate compared with free-drug gel, indicating formation of a long-lasting cutaneous depot.	Prolonged anti-edematous and analgesic effects in rat paw inflammation models, maintaining inhibition of swelling longer than conventional piroxicam gel.	Occlusive, lipid-rich nanocarrier film increases partitioning of piroxicam into epidermis and periarticular tissue while restricting back-diffusion into systemic circulation, attenuating systemic exposure.	Feasible chronic OA pain management with reduced oral piroxicam requirement, thereby lowering the risk of GI and renal complications while sustaining local symptom control.	Krishnatreyya et al. (2019); Peng et al. (2017)
Ketorolac	Osteoarthritis-associated acute inflammatory pain	Conventional ketorolac tromethamine gel displays rapid drug release (~ 86% in 24 h) and a relatively short-lived anti-inflammatory effect in edema models.	Ketorolac-loaded SLN gels using stearic acid–based lipid cores incorporated into carbopol bases.	Slower release (~ 55% at 24 h) and more controlled diffusion from the gel, with increased residence in cutaneous tissue versus conventional gel.	Higher maximal edema inhibition (~ 79% at 6 h) and measurable anti-inflammatory effect persisting to 24 h compared with conventional gel in carrageenan-induced paw edema.	Solid lipid core entraps ketorolac, acting as a sustained-release depot that maintains therapeutic concentrations at the inflamed site while avoiding high systemic peak levels associated with toxicity.	Longer duration of pain relief from a single application and potential reduction in dosing frequency, supporting safer use of potent NSAIDs in OA-related pain.	Bhatia and Gupta (2016); Khoware et al. (2021)
Methotrexate	Rheumatoid arthritis and rheumatic joint diseases	Oral or parenteral methotrexate and intra-articular solution exhibit variable absorption, first-pass hepatic metabolism, rapid clearance from joints, and dose-limiting hepatic and hematologic toxicity.	Methotrexate-loaded nanostructured lipid carrier (SLN-type) thermo-sensitive intra-articular smart gel with ~ 100 nm particles and ~ 70% entrapment.	Extended drug release over approximately 108 h and prolonged joint residence compared with free MTX, with reduced systemic spillover after intra-articular injection.	Marked and sustained reduction in joint swelling and histological inflammation for up to 28 days in adjuvant arthritis rats, outperforming intra-articular MTX solution.	Intra-articular lipid depot gradually liberates MTX directly into synovial tissue, maintaining local therapeutic levels while minimizing systemic exposure and bypassing hepatic first-pass pathways.	Enhanced local control of RA-related synovitis with potential for lower cumulative MTX dosing and decreased risk of systemic toxicities.	Mello et al. (2013); Boechat et al. (2015)
Prednisolone	Rheumatoid arthritis	Oral and IV prednisolone provide strong systemic immunosuppression but have relatively short half-life and cause metabolic, cardiovascular and skeletal adverse effects with chronic high-dose use.	Hyaluronic acid-coated prednisolone SLNs (HA–SLNs) for IV administration in collagen-induced arthritis; ~200 nm particles with CD44-targeting surface.	Prolonged circulation and preferential accumulation in inflamed joints compared with non-targeted SLNs and free prednisolone, resulting in higher joint/plasma concentration ratios.	Greater suppression of TNF-α, IL-1β and IL-6, improved arthritis scores and preservation of bone and cartilage versus equivalent doses of free prednisolone.	HA shell binds CD44 receptors overexpressed on activated synoviocytes and macrophages, concentrating prednisolone within inflamed synovium and reducing exposure of non-inflamed tissues.	Enhanced anti-arthritic efficacy with reduced systemic steroid burden, supporting the concept of steroid-sparing targeted nanotherapy in RA.	Zhou et al. (2018)
Curcumin	Rheumatoid arthritis and inflammatory joint disease	Conventional oral curcumin exhibits extremely low bioavailability due to poor aqueous solubility, extensive intestinal/hepatic metabolism and rapid elimination, limiting clinical utility despite potent anti-inflammatory activity.	Curcumin-loaded SLNs for oral or transdermal delivery (200–300 nm), including binary lipid systems and ceramide–palmitic acid matrices.	Several-fold increase in systemic exposure and improved tissue distribution after oral dosing, and markedly enhanced skin permeation for transdermal systems compared with free curcumin.	In CFA-induced arthritis, curcumin-SLNs significantly outperform free curcumin at equivalent doses in reducing paw swelling, hyperalgesia, oxidative–nitrosative stress, inflammatory cytokines and radiological joint damage.	Encapsulation enhances solubility and protects curcumin from rapid metabolism, while nanoparticle uptake by immune and joint cells allows more effective modulation of NF-κB and cytokine pathways at the inflamed site.	Robust amelioration of experimental arthritis at lower curcumin doses, indicating improved therapeutic index and feasibility as an adjunct anti-inflammatory therapy in RA.	Jj and Raja Lakshmi (2017), Gaur et al. (2016)

## Patents and marketed lipid nanoparticle products in anti-inflammatory drug delivery

Many patents have disclosed nanoformulations (lipid-based nanoparticles and solid lipid nanoparticles) to enhance the pharmacological activity of anti-inflammatory medicines. These patented systems include SLNs, liposomes, nanoemulsions, and other lipid nanocarriers created for NSAIDs, corticosteroids, disease-modifying antirheumatic drugs, and natural anti-inflammatory compounds used in the treatment of osteoarthritis, rheumatoid arthritis, and chronic inflammatory diseases. Table 5 is a summary of selected representative patents in anti-inflammatory drug delivery.

Despite high patent activity and promising preclinical outcomes, very few lipid-based nanomedicine products have reached the market. Most licensed formulations of nanoparticles are utilized for oncology, infectious diseases, anesthesia, and vaccination but not for inflammatory or arthritic disorders. Only a limited number of marketed nanoemulsion and liposomal topical or ocular inflammatory products exist, whereas yet no solid lipid nanoparticle formulation has received approval for osteoarthritis or rheumatoid arthritis. The disconnect between experimental research and patent generation on the one hand and clinical approval on the other illustrates the challenges associated with long-term safety, large-scale manufacturability, and regulatory constraints for lipid nanoparticle-based drug delivery systems.

**Table 5:** Representative patented lipid nanoparticle and solid lipid nanoparticle drug delivery systems for anti-inflammatory agents in osteoarthritis, rheumatoid arthritis, and related inflammatory disorders

Disease area / use case	Drug class	Example compound(s)	Nanoparticle system (type)	Representative patent(s)	Notes on indication / use
Osteoarthritis, joint and back pain	NSAID	Ibuprofen	Solid lipid nanoparticles in topical gel prepared by hot-melt extrusion	Sachdeva et al. (2021) US11135176B1	Topical SLN gel for local treatment of inflammatory pain and musculoskeletal conditions; continuous HME manufacturing.​
Osteoarthritis, joint and back pain	NSAID	Diclofenac (salt + free acid)	Nanoemulsion with triglyceride lipid phase and non-ionic surfactant (lipid nanoparticles)	Alonso et al. (2025) US20250262178A1	Transparent/self-emulsifying nanoemulsion for topical treatment of joint pain, osteoarthritis, muscle pain, back pain, and inflammation.​
Osteoarthritis, joint and back pain	NSAID	Diclofenac alkali metal salt	Nanocomplex with glycol, C2–C3 alcohol, cationic surfactant (nanostructured lipid-rich system)	Alonso et al. (2024) US20240325333A1	Topical nano-complex for joint pain, osteoarthritis, muscle pain, back pain, and inflammation with high permeation and favorable zeta potential.​
Rheumatoid arthritis, autoimmune diseases	Conventional DMARD	Methotrexate (alpha- or gamma-polyglutamated)	Pegylated or non-pegylated liposomes (20–500 nm) encapsulating polyglutamated MTX	Niyikiza and Moyo (2021) US20210154196A1	Liposomal MTX derivatives proposed for hyperproliferative and immune disorders including rheumatoid arthritis, with altered PK and tissue distribution.
Rheumatoid arthritis, autoimmune / inflammatory diseases	Natural anti-inflammatory	Curcumin / curcuminoids	Curcumin–cyclodextrin complex formulated as solid lipid nanoparticles or related colloidal systems	Desai (2010) US20100179103A1	Curcumin–cyclodextrin complex used in SLNs, microemulsions, or gels for autoimmune and inflammatory diseases including rheumatoid arthritis.​
Anti-arthritic and chronic inflammatory indications (preclinical)	Natural anti-inflammatory	Curcumin	Solid lipid nanoparticles (20–800 nm) prepared by high-pressure homogenization	Kaur et al. (2022) US20220151945A1	Curcumin SLN process; background emphasizes anti-arthritic and anti-rheumatoid arthritis activity of curcumin as motivation for improved delivery.​
Pain and inflammatory disorders (including rheumatic problems)	Corticosteroid	Dexamethasone / dexamethasone sodium phosphate	Multivesicular liposomes (MVL) with multiple aqueous chambers and lipid membranes	Davis et al. (2023) US20230137783A1	Injectable MVL formulations providing 3–28 days extended release of dexamethasone for treatment of pain and inflammation, including rheumatic conditions.​
Broad inflammatory and degenerative disorders	Natural anti-inflammatory / nutraceuticals	Curcumin and other polyphenols or phytochemicals	General solid lipid nanoparticle platform encapsulating diverse bioactive compounds	Baek et al.(2025) US20250302764A1	SLN platform encapsulating vitamins, polyphenols, and phytochemicals to improve bioavailability; applicable to natural anti-inflammatory agents like curcumin.​
Chronic inflammatory and degenerative diseases	Natural anti-inflammatory	Curcumin	Solid lipid particles (0.1–10 μm) with lecithin, glycerol behenate, and glycerides	Diorio and Lokhnauth (2019) US10166187B2,	Oral solid lipid particles designed to enhance curcumin’s stability and bioavailability for systemic anti-inflammatory and antioxidant therapy.
Broad inflammatory disorders	Corticosteroid / lipophilic anti-inflammatory platform	Budesonide esters and other lipophilic anti-inflammatory agents	Lipid nanoparticles	Lindfors and Kjellman (2017) WO2017194454A1	distinct anti-inflammatory LNP platform using a lipid phase with lipophilic anti-inflammatory agents; not a duplicate of your diclofenac, methotrexate, or curcumin U.S. families

## Clinical translation gap: why SLNs have not yet reached routine clinical use

Despite promising preclinical results, the majority of SLN-based anti-inflammatory formulations are reliant on short-term rodent studies and in vitro models, which fail to accurately represent the long-term progression of osteoarthritis and rheumatoid arthritis. Standard evaluation metrics such as 24-hour dermal retention for topical NSAIDs or approximately 108-hour joint persistence following a single intra-articular administration—indicate pharmacokinetic viability but do not adequately address the complexities of flare-remission cycles, interactions with concomitant medications, and adherence challenges that are critical for chronic management. Even advanced targeted systems (e.g., HA-coated prednisolone; albumin-lipid dexamethasone palmitate) predominantly consist of one-time treatment studies, resulting in unresolved inquiries regarding repeated dosing, immunological memory, and potential for disease modification.

A secondary challenge lies in the scarcity of long-term pharmacokinetic, biodistribution, and safety data under chronic administration. Sustained joint retention and RES sequestration may provide benefits or lead to unpredictable accumulation in the liver, spleen, and inflamed synovium; however, studies addressing multi-month mass balance, metabolite profiling, and immunotoxicology are infrequent (Shinde et al. 2016; Zhou et al. 2018; Dong et al. 2024). Simultaneously, the transition to manufacturing presents significant complexities: managing particle size (approximately 100–200 nm), polymorphic transitions, and drug release during storage, while maintaining release kinetics after scaling and sterilization, poses challenges to batch consistency and shelf-life assertions (Medina-Montano et al. 2022).

Finally, the regulatory pathways for nanomedicines necessitate a more detailed characterization than traditional small molecules specifically, stringent control of critical quality attributes (size distribution, crystallinity, surface chemistry), diverse release methodologies, stability under practical handling conditions, and comparability following process modifications. The transition from exploratory to commercial production, establishing in vitro-in vivo correlation (IVIVC) for intricate carriers, and defining bioequivalence for targeted or depot characteristics remain evolving challenges that extend timelines and increase costs. Moving forward, clinically viable SLN initiatives will necessitate chronic, multidose pharmacokinetic/biodistribution studies, Quality by Design (QbD)-informed scaling with fidelity to release profiles, and proactive engagement with regulatory bodies to ensure alignment on Chemistry, Manufacturing, and Controls (CMC) expectations specific to arthritis treatments (Zhou et al. 2018; Zheng et al. 2024).

## Regulatory considerations for nanomedicine

SLNs constitute one category of nanomedicine-based drug delivery systems, and thus their formulations require more regulatory assessment than classical formulations. Nanoparticle properties can drastically affect pharmacokinetics, biodistribution, safety, or therapeutic function, and therefore regulatory bodies [such as the U.S. Food and Drug Administration (FDA), European Medicines Agency (EMA), Central Drugs Standard Control Organization (CDSCO)] recommend determining whether products that utilize nanotechnology should be assessed case by case. In contrast to traditional dosage forms, SLN formulations usually need a very extensive physicochemical characterization; assessment of nanoparticle-specific critical quality attributes, and for chronic diseases like osteoarthritis and rheumatoid arthritis, such detailed long-term toxicity studies are mandatory (Medina-Montano et al. 2022; Zheng et al. 2024). Moreover, the need for manufacturing reproducibility, stability, and scale-up is a critical regulatory issue with lipid nanoparticle systems. The key regulatory requirements of SLN-based formulations are summarized in Table 6.

**Table 6:** Regulatory requirements for SLN / nanomedicine formulations compared with conventional drug products (U.S. Food and Drug Administration 2017)

Category	Conventional formulation requirement	Additional requirement for SLN / nanomedicine	Regulatory significance
Particle size	Not mandatory	Particle size, size distribution, morphology (TEM/SEM/DLS)	Influences biodistribution and clearance
Surface properties	Not required	Zeta potential, surface chemistry, coating	Affects stability and cell interaction
Composition	API + excipients	Lipid matrix, surfactant, stabilizer, coating material	Needed for reproducibility
Structural characterization	Limited	Crystallinity, polymorphism, lipid phase behavior	Determines drug release
Drug loading	Assay only	Entrapment efficiency + loading capacity	Needed for dose accuracy
Drug release	Dissolution	In-vitro release + controlled release profile	Predicts in-vivo behavior
Stability	Chemical stability	Physical + chemical + aggregation stability	Shelf-life determination
Pharmacokinetics	Standard PK	PK + biodistribution + tissue accumulation	Required for nanomedicine
Toxicity	Acute + chronic	Immunotoxicity, RES uptake, organ deposition	Important for chronic use
Long-term safety	Limited	Repeated dose, chronic exposure studies	Required for arthritis therapy
Manufacturing	GMP	Critical process parameters (CPP, CQA, QbD)	Ensures batch consistency
Scale-up	Standard	Process validation for nanoparticle systems	Needed for approval
Regulatory pathway	NDA / ANDA	Case-by-case evaluation	No universal nanomedicine guideline
Clinical studies	Standard	Additional safety + PK monitoring	Required for nano-products

These added regulatory considerations underscore the complexity of developing SLN-based anti-inflammatory formulations relative to conventional dosage forms. Changes in particle size, lipid composition, or manufacturing conditions can have a profound impact on drug release, biodistribution, and safety profile of the carrier, and thus critical quality attribute needs to be stringently controlled. Hence, the regulatory agencies recommend a Quality-by-Design approach, extensive characterization, and long-term pharmacokinetic and toxicity studies prior to approval of nanomedicine products. While regulatory frameworks for lipid nanoparticles are being developed, the establishment of standardised evaluation criteria will be critical to enabling the clinical translation of SLN-based therapies for chronic inflammatory disease.

## Future perspectives

Since these SLNs encapsulate drug strategies with lipid matrix that is biocompatible, it enhances drug solubility, protects sensitive compounds while facilitating a continuous or specific release which theoretically makes them potential delivery systems for upcoming anti-inflammatory treatment. However, the clinical translation of SLNs has been impeded by ongoing issues, including batch-to-batch variability when manufacturing SLNs, and drug expulsion during storage, as well as lipid polymorphic transitions, alongside a limited understanding of the link between critical quality attributes and in vivo performance. Successful clinical development will depend on overcoming these challenges through more robust formulation approaches, standardized characterization techniques, and scalable manufacturing processes (Ahuja and Rajawat 2021; Pandey et al. 2022).

SLNs may hold particular promise for targeted therapy in rheumatoid arthritis; infiltrating CD44(+) cells that aggregate in inflamed synovial tissue may serve as carriers for this drug delivery system. Certain surface-modified SLNs, for example hyaluronic acid-coated prednisolone nanoparticles, have exhibited increased joint accumulation and superior anti-inflammatory potency as compared with free drug in various experimental models. Future SLN systems could also be engineered to deliver disease-modifying antirheumatic drugs, glucocorticoids, JAK inhibitors, or nucleic acid-based therapeutics, thereby reducing systemic toxicity while retaining therapeutic effects (Albuquerque et al. 2015; Zhou et al. 2018; Upadhyay and Soni 2025).

The SLNs formulations targeting intra-articular delivery is an important research orientation for osteoarthritis. Standard intra articular injections are cleared quickly from the joint, with poor penetration into cartilage. For example, surface-engineered nanoparticles that can interact with cartilage matrix components or collagen may have a longer residence time in the joint and potentially improve drug distribution in the tissue. Such systems could function as long-acting depots for corticosteroids, NSAIDs, senolytic agents or disease-modifying drugs and may improve the longevity of osteoarthritis treatment (Pandey et al. 2022; Wen et al. 2023).

The future of SLN-based therapies will also critically depend on regulatory considerations. Current regulatory recommendations for nanomedicines detail evaluation of particle size, morphology, surface characteristics and stability, release behavior and manufacturing reproducibility. Thus, most new studies should assess not just pharmacological efficacy but also develop appropriate quality control measures, provide long-term safety data and generate well-designed clinical trials. Further development of SLNs to ensure biocompatibility, drug stability with embedded therapeutic agents or biomolecules, and tunable targeting strategies could let them grow from experimental nanocarriers into a relevant platform for precision-mediated therapy in RA, OA, and chronic inflammatory diseases.

## Conclusion

SLNs offer a promising approach to enhancing the effectiveness and tolerability of potent anti-inflammatory drugs for chronic conditions such as osteoarthritis and rheumatoid arthritis. As discussed in this paper, SLNs systems significantly influence key pharmacokinetic properties, including absorption, bioavailability, release kinetics, tissue distribution, and residence time. These factors lead to increased local drug exposure while reducing systemic exposure. This reduction is particularly beneficial in addressing common pharmacological issues associated with traditional anti-inflammatory agents, including a short half-life, lack of tissue selectivity, and dose-limited toxicity. Importantly, SLNs have a substantial pharmacodynamic effect and serve not only as drug carriers but also as active modulators of anti-inflammatory pharmacology. Incorporating drugs into solid lipid matrices enhances their inhibitory effects against key inflammatory mediators and signaling pathways, including TNF-α, IL-1β, IL-6, COX-2/PGE₂, NF-κB, and JAK/STAT pathways.

Targeted or surface-modified SLN systems improve drug delivery to inflamed tissues, particularly in the synovial joint, further confirming their role in active modulation rather than merely serving as carriers. The preclinical toxicological profiles of SLNs are generally favorable, largely due to the use of non-toxic materials in lipid excipients, their biodegradability, and the dose-reduction effect achieved through targeted delivery. However, much of the current knowledge is based on short-term animal and in vitro studies, while long-term pharmacokinetics, biodistribution, and immunological effects under chronic dosing conditions remain inadequately addressed. Additionally, challenges related to formulation stability, large-scale manufacturing reproducibility, and the regulatory definition of nanoparticle-specific quality attributes still pose barriers to clinical translation. SLNs represent a rational tool to enhance the therapeutic index of anti-inflammatory drugs by combining pharmacokinetic improvements with targeted intervention in inflammatory pathways. Compared with conventional formulations, SLN-based anti-inflammatory systems offer important advantages in terms of controlled release, improved bioavailability, enhanced local retention, reduced systemic exposure, and better targeting of inflamed tissues. The growing patent activity in this area further reflects the translational potential of these systems. However, their clinical application remains limited by insufficient long-term safety data, manufacturing and scale-up challenges, and evolving nanoparticle-specific regulatory requirements. Therefore, future studies should prioritize repeated-dose evaluation, standardized quality control, and well-designed clinical validation in osteoarthritis and rheumatoid arthritis. Nevertheless, the transition to routine clinical use relies on comprehensive studies of chronic exposure, standardized pharmacological evaluations, and manufacturing processes that meet regulatory requirements for complex nanomedicines. Meeting these requirements will be essential to unlock the potential of SLNs as next-generation anti-inflammatory agents.

## Data Availability

Data sharing is not applicable to this article as no new datasets were generated or analyzed during the current study.
